# Evaluation of antioxidant, anti-inflammatory, analgesic and antipyretic activities of the stem bark of *Sapindus mukorossi*

**DOI:** 10.1186/s12906-017-2042-3

**Published:** 2017-12-08

**Authors:** Madeha Shah, Zahida Parveen, Muhammad Rashid Khan

**Affiliations:** Department of Biochemistry, Faculty of Biological Sciences, Quaid-i-Azam University, Islamabad, Pakistan

**Keywords:** Anti-inflammatory, Analgesic, Antipyretic, Antioxidant, *Sapindus mukorossi*

## Abstract

**Background:**

Saponins are the main constituents of genus *Sapindus* and have the therapeutic potential for inflammatory disorders. In this study the antioxidant, anti-inflammatory, analgesic and antipyretic potential of the stem bark of soap nut (*Sapindus mukorossi*) methanol extract and its derived fractions has been investigated.

**Methods:**

Powder of stem bark of the *S. mukorossi* was extracted with methanol (SMM) and fractionated in order of n-hexane (SMH), chloroform (SMC), ethyl acetate (SME), n-butanol (SMB) and the remaining as aqueous fraction (SMA). Quantitative estimation for the total phenolic and total flavonoid content was carried out in all the extract/fractions. Further, various in vitro antioxidant assays were also performed. Anti-inflammatory (Carrageenan induced paw edema), analgesic (hot plate latency test) and antipyretic (rectal temperature) were determined in Sprague-Dawley rat.

**Results:**

Quantitative estimation of total phenolic contents in extract/fractions varied between 252.3 ± 2.41 mg of GAE/g - 594.16 ± 4.3 mg of GAE/g while the total flavonoids estimated were from 11.02 ± 1.3 mg of RUE/g to 96.9 ± 3.2 mg of RUE/g. Standard antioxidant assays such as scavenging of DPPH, hydroxyl radical, nitric oxide, phosphomolybdenum assay, reducing power, inhibition of β-carotene bleaching, iron chelation activity and inhibition of heat induced protein denaturation indicated the antioxidant potential of the extract/fractions. Carrageenan induced paw edema of rat was effectively inhibited by SMA at 300 mg/kg administration to rat (84.19 ± 1.48%) after 3 h and analgesia (latency time) in hot plate test (55.78 ± 1.22%) after 120 min. SMA at 300 mg/kg distinctly decreased the rectal temperature in brewer’s yeast (*Saccharomyces cerevisiae*) induced pyrexia in rat.

**Conclusion:**

The resulted obtained in this study suggested the therapeutic importance of stem bark of *S. mukorossi* in inflammatory related disorders.

## Background

It is demonstrated that most of the ailments are because of the oxidative stress, condition developed as a result of excessive generation of free radicals to that of the physiological requirement of the body. Low levels of antioxidants in the living system assist the development of ageing related ailments including atherosclerosis, cancers, diabetic neuropathy, Alzheimer’s disease and inflammatory disorders. Biological macromolecules such as proteins, lipids and DNA are damaged by the deleterious action of free radicals. Presence of enzymes like superoxide dismutase, catalase and/or compounds such as ascorbic acid, tocopherol and glutathione scavenged the free radicals and thus provide a shield against the damaging action of oxidative stress [1].

Inflammation is the sensitive state of hyperemia from blood vessels with consequential redness, warmness, swelling and discomfort that tissue displays in reaction to corporal or biochemical injury [2]. It is attained by the migration of plasma and white cells comprising monocytes that are locally distinguish into macrophages from blood into wounded tissues. Immune reaction is vital for the body to remove dangerous pathogens and it is categorized by way of an acute inflammation. The determination of inflammation involves the dissolution of pro-inflammatory signaling trails and allowances the re-establishment of normal tissue task. A failure of these processes can lead to chronic inflammation. In inflammation various pro- and anti-inflammatory intermediates are produced comprising cytokines, interleukins, ROS, chemokine’s all of them play serious role in governing the inflammation [3]. Dysregulation of the immune responses can lead to various acute and chronic inflammatory disorders. Clinically inflammatory disorders are usually managed by steroidal (betamethasone) and the non-steroidal anti-inflammatory drugs (NSAIDs; acetylsalicylic acid), however, chronic administration of such drugs can cause gastrointestinal, renal and cardiovascular disorders [4].

Pain is a well-defined as an unwanted corporeal or demonstrative experience. And it is also categorized as acute or chronic pain. There are numerous forms of painkillers that are categorized into three groups: opioid analgesics (morphine and codeine), non-opioid analgesics (NSAIDs; aspirin, diclofenac) and adjuvant analgesics, which are composites generally taken for purposes other than pain, but could be taken in some circumstances. Antidepressant medicines can use as pain reliever in the cure of various chronic discomfort states [5].

Pyrexia is an increase in body temperature beyond normal biological range that may be due to biological stress such as in the course of ovulation, amplified thyroid secretion, too much exercise, any abrasions to central nervous system, owing to leukemia and frequently in microbial infections. The contagious agent or injured tissues recruit the proliferation of pro-inflammatory facilitator’s cytokines (IL-1 β) and TNF-α that increase the development of prostaglandin (PG)E_2_ close to the gastric hypothalamus region and the prostaglandin in return act on the hypothalamus to increase the body temperature. The higher body temperature is decreased by antipyretic medications which inhibit COX-2 enzyme expression that prevented the prostaglandin production. Conversely these man-made antipyretic agents prevent the COX-2 with great selectivity but on the other hand they have lethal effects on other organs of body that is glomeruli, cortex of the brain, hepatic cells and cardiac muscles, however plant based COX-2 inhibitors have lesser selectivity with rarer harmful effects [6].

An anticipated solution to such problem is the enhancement of antioxidants through ingestion of natural antioxidants in the form of vegetables, fruits and various medicinal plants. Antioxidants either scavenge the ROS or reduce the generation of free radicals which can serve as a defensive medicine for a number of diseases [7]. Phyto-constituents carried out their antioxidant function either by scavenging or termination of chain reactions controlled by ROS or by forming a resistant shield to defend the antioxidant protection mechanism. On account of various categories of free radicals diverse methodologies have been used to establish the in vitro antioxidant potential of the extracts/substances. There is a complex mechanism of scavenging free radicals inside human body and consists of a group of enzymes. Consequently different assays have been planned to get a complete picture of antioxidant potential of medicinal plants [8].

*Sapindus mukorossi* Gaertn. of family Sapindaceae is extensively distributed in the upper ranges of Indo-Gangetic grasslands, Shivaliks and the sub Himalayan range. It is ordinarily known as Aritha or Ritha. It is a deciduous tree by a straightforward trunk about 12 m in height. *S. mukorossi* is well known for its traditional therapeutic standards. The fruits are of considerable importance for their medicinal value for treating a number of diseases like excessive salivation, pimples, epilepsy, chlorosis, migranes, eczema and psoriasis [9]. The powdered seeds are employed in the treatment of dental caries, arthritis, common colds, constipation and nausea [10]. Bark of root and fruits of *S. saponaria* has important role in medicine and are used as tranquilizer, astringent, diuretic, expectorant, tonic, blood cleanser, healing and to counter cough [11]. Seeds are used in the cure of dental caries, arthritis, common colds, constipation and also in nausea [12]. According to Albiero et al. [11] fruit is used for curing ulcers, external wounds and inflammation. Leaves of *S. mukorossi* are used in baths to get rid of joint discomfort and the roots are also used in the cure of the gout and rheumatism [12]. In genus *Sapindus* saponins and acyclic sesquiterpene oligoglycosides are found to be the main phytoconstituents exerting various pharmacological activities [13]. Abdel-Wahab and Selim [14] detected the presence of carbohydrates, steroids, flavonoids, and saponins in leaves and stems of *S. saponaria*. From the fruit of *S. saponaria* anti-inflammatory and antioxidant compounds stigmasterol, oleanolic acid, luteolin, luteolin 8-퐶-훽-glucoside (orientin), luteolin 6-퐶-훽-glucoside (isoorientin), luteolin 7-푂-훽-glucuronide, and rutin have been isolated [15]. Antioxidant effects of *S. mukorossi* extract against CCl_4_ induced liver cirrhosis have studies in rat [16]. Extract obtained from the fruit of *S. mukorossi* strongly exhibited anti-hyperglycemic and anti-hyperlipidemic activities in streptozotocin induced diabetic rats. The extract was also able to restore the haematological and histopathological changes of pancreas towards the normal rats [17]. Jedage and Manjunath [18] studied the nephroprotective activity of the extract derived from the bark of *S. emarginatus* in rat. Extract of *S. mukorossi* exhibited the anxiolytic activity in mice [19] whereas the antiepileptic activity has been studied in rat [20]. Chen et al. [21] investigated the tyrosinase inhibition, free radical scavenging, antimicroorganism and anticancer proliferation activities of *S. mukorossi* extracts. Anti-inflammatory effects of the leaves of *S. mukorossi* and *S. laurifolius* have been studied for carrageenan induced paw edema in rat [22, 23]. Arulmozhia et al. [24] investigated the effects of *S. trifoliatus* for various migraine targets during in vitro studies. Antihyperalgesic activities of the leaf extract of *S. trifoliatus* were reported in earlier studies in rat [25]. Phytoconstituents such as hederagenin and crude saponin isolated from *S. mukorossi* exerted anti-inflammatory activity in carrageenan induced paw edema, granuloma pouch and adjuvant induced arthritis in rat. Antinociceptive properties have also been investigated for acetic acid induced writhing test in rat. [26]. Antinociceptive and anti-inflammatory effects of the saponin and sapogenins obtained from the stem of *Akebia quinata* have also been recorded [27]. Saponins and flavonoids of *Zizyphus lotus* inhibited paw edema and hyperalgesia in mice and nitrite production in lipopolysaccharide (LPS) stimulated RAW 264.7 macrophages without affecting cell viability [28]. Arraua et al. [29] studied that *Quillaja saponaria* bark constituted of mainly triterpene saponins and has exhibited more antinociceptive aptitude than ibuprofen in murine thermal models. Saponin fraction of *Nigella glandulifera* seeds exerted strong anti-inflammatory, analgesic and antioxidant potential in mice [30]. Anti-inflammatory and antinociceptive activities of various plants constituting high concentration of saponins have been reported [28, 31–36]. The *S. mukorossi* contained high percentage of saponins [37, 38]. In this investigation methanol extract of stem bark of *S. mukorossi* and its derived fractions were subjected to different assays for evaluation of its antioxidant, anti-inflammatory, analgesic and antipyretic activities in rat. The methanol extract and the derived fractions were also subjected for the presence of various phytochemical classes.

## Methods

### Plant collection

Collection of *S. mukorossi* Gaertn. stem bark was carried out from Islamabad in month of March to April in 2016. It was recognized by Dr. Muhammad Zafar of Department of Plant Sciences and an accession number (175621) was given by Herbarium of Pakistan, Quaid-i-Azam University Islamabad, Pakistan.

### Extract preparation

For three weeks stem bark of *A. nitida* was shade dried and ground with electric grinder. Twice extraction of 2 kg powder was carried out in 8 l of the commercial methanol for one week. Filtrate obtained was dried under vacuum in a rotary evaporator at 40 °C to obtain the viscous material (SMM) that stored at 4 °C. Fractionation /was done distinctly to attain the compounds from crude extract conferring to increase/in the polarity. An amount of 50 g of SMM was suspended in 200 ml of distilled water/and distributed to liquid-liquid partition. The scheme of solvents used was in order of n-hexane (SMH), chloroform (SMC), ethyl acetate (SME), n-butanol (SMB) and the remaining soluble portion as aqueous (SMA). Each fraction was dried as above stored at 4 °C for various studies.

### Quantitative phytochemical assessment

#### Total phenolic contents

Determination of total phenolic contents in the samples was performed according to Kim et al. [39]. For this purpose 1 ml of each sample (1 mg/ml) was mixed with 9 ml of deionized water followed by addition of 1 ml of Folin-Ciocalteu’s phenol reagent and allowed to stand for 5 min. Then 10 ml of 7% Na_2_CO_3_ solution and 13 ml of deionized water was added to the reaction mixture. After keeping the reaction mixture at 23 °C for 90 min the absorbance was ensured at 750 nm. The calibration curve of gallic acid was used for the calculation of the total phenolic contents. Assay was performed in triplicate. Quantity of total phenol contents in plant samples was expressed as milligram of gallic acid equivalents per gram of dried sample.

#### Total flavonoid contents

The spectrophotometric technique was used to estimate the total flavonoid contents in the extract/fractions [40]. The reaction mixture was prepared by the addition of 0.3 ml of plant sample to a mixture containing 0.15 ml of NaNO_2_ (0.5 M), 0.15 ml of AlCl_3_.6H_2_O (0.3 M) and 3.4 ml of methanol (30%). The mixture was kept for 5 min and 1 ml of NaOH (1 M) was mixed in it. The absorbance of the reaction mixture was recorded at 506 nm. Total flavonoid contents were estimated by using the rutin as standard. The total flavonoid contents were expressed as milligram of rutin equivalent per gram of sample.

### In vitro antioxidant studies

#### DPPH (1, 1-diphenyl-2-picryl-hydrazyl) radical scavenging assay

The DPPH scavenging potential of various plant samples was estimated according to the method of Mensor et al. [41]. DPPH solution (900 μl) having absorbance of 0.908 (± 0.02) at 517 nm was mixed under dark with 100 μl of plant samples at various concentrations. After placing the reaction mixture for 15 min at room temperature, absorbance was recorded at 517 nm. DPPH radical scavenging activity of the plant sample was determined by following Eq. 1.

$$ \mathrm{Scavenging}\  \mathrm{effect}\ \left(\%\right)=\left(\frac{\mathrm{Control}\  \mathrm{absorbance}-\mathrm{Sample}\  \mathrm{absorbance}}{\mathrm{Control}\  \mathrm{absorbance}}\right)\times 100 $$

#### Hydroxyl radical scavenging assay

The hydroxyl free radical scavenging ability of the plant samples was estimated according to the methodology of Halliwell et al. [42]. The reaction mixture contained 500 μl of 2-deoxyribose (2.8 mM) in phosphate buffer (50 mM; pH 7.4), 0.1 M of EDTA (1:1; *v*/v), 200 μl of ferric chloride (100 mM), 100 μl of H_2_O_2_ (200 mM) and 100 μl of plant sample. The initiation of reaction was brought by the introduction of 100 μl of ascorbic acid (300 mM) and incubated for 1 h at 37 °C. Then 1 ml of trichloroacetic acid (2.8%) and 1 ml of aqueous thiobarbituric acid (1%; *W*/*v*) prepared in NaOH (50 mM) were added to the reaction mixture. The whole recipe was heated for 15 min in a boiling water bath and cooled down to room temperature. The absorbance of the reaction mixture was recorded at 532 nm. The hydroxyl radical scavenging activity of the sample was estimated by the following formula:

$$ \mathrm{Scavenging}\  \mathrm{effect}\ \left(\%\right)=\kern0.5em \left(\frac{1-\mathrm{Sample}\  \mathrm{absorbance}}{\mathrm{Control}\  \mathrm{absorbance}}\right)\times 100 $$

#### Nitric oxide scavenging assay

The method developed by Leone et al. [43] to estimate the nitric oxide scavenging activity of the plant sample was used in this study. The Griess reagent was prepared by mixing equimolar quantity of 0.1% napthylenediamine in distilled water and 1% of sulphanilamide in 5% phosphoric acid. An aliquot of 100 μl of the plant sample was mixed with 100 μl of sodium nitroprusside (10 mM) in saline phosphate buffer and 1 ml of the Griess reagent. After keeping the reaction mixture for 3 h at room temperature the absorbance was recorded at 546 nm. Scavenging potential of the extract was determined by Eq. 1.

#### Chelating power assay

The chelation ability of the plant sample for iron (II) was estimated by following the protocol of Robinson et al. [44]. An aliquot of 200 μl of various concentrations of the plant sample was mixed with 900 μl of methanol and 100 μl of FeCl_2_.2H_2_O (2.0 mM) and kept it at room temperature for 5 min. The reaction was initiated by adding 400 μl of ferrozine (5 mM) and kept for 10 min at room temperature. The absorbance of the reaction mixture was noted at 562 nm using EDTA as standard in comparison. The chelating power was determined by Eq. 1.

#### Inhibition of β-carotene bleaching assay

The ability of plant sample to inhibit the β-carotene bleach was investigated according to Dapkevicius et al. [45]. A quantity of 2 mg of β-carotene was dissolved in 10 ml of chloroform followed by mixing of 200 mg of tween 80 and 20 mg of linoleic acid. The chloroform was evaporated under vacuum and then 50 ml of distilled water was added to it, vigorously mixed to get a uniform emulsion of β-carotene linoleate. An aliquot of 30 μl plant sample was added to 250 μl of the freshly prepared emulsion and absorbance at 470 nm was recorded at 0 h and after 2 h of incubation at 45 °C. Catechin served as standard in this assay. β-Carotene bleaching inhibition was estimated by this formula.

$$ \mathrm{Bleaching}\  \mathrm{inhibition}\ \left(\%\right)=\kern0.5em \left[\left({\mathrm{A}}_{0\mathrm{t}}\hbox{--} {\mathrm{A}}_{120\mathrm{t}}\right)\kern0.5em /\kern0.5em \left({\mathrm{A}}_{0\mathrm{c}}\hbox{--} {\mathrm{A}}_{120\mathrm{c}}\right)\right]\kern0.5em \times 100 $$

#### Reducing power assay

By the method of Fejes et al. [46] reducing power activity of the plant samples was determined. An aliquot of 2 ml of plant sample was mixed with 2 ml of phosphate buffer (0.2 M; pH 6.6) and 2 ml of potassium ferricyanide (10 mg/L). After incubation of the reaction mixture at 50 °C for 20 min 2 ml of trichloroacetic acid (TCA) (100 mg/L) was added to the reaction mixture. A volume of 2 ml of the reaction mixture was diluted with 2 ml of distilled water and then 0.4 ml of FeCl_3_ (0.1%) was added to the test tube. Absorbance of the reaction mixture was determined at 700 nm after keeping the reaction mixture for 10 min at room temperature.

#### Total antioxidant activity (Phosphomolybedenum assay)

The antioxidant capabilities of the plant sample were assured by the phosphomolybdenum assay as per described in the methodology of Umamaheswari and Chatterjee [47]. The plant sample (0.1 ml) was mixed with the reagent solution composed of 28 mM of Na_3_PO_4_, 0.6 M of H_2_SO_4_ and 4 mM of ammonium molybdate. After heating the reaction mixture at 95 °C in water bath for 90 min under dark conditions was cooled to room temperature and absorbance was measured at 765 nm.

### In vitro anti-inflammatory activity

Anti-inflammatory potential of the stem bark of *S. mukorossi* Gaertn*.* was studied according to the method of Sakat et al. [48]. Reaction mixture comprised of 900 μl of bovine serum albumin (BSA) and 100 μl of plant sample of varied concentrations. The reaction mixture was incubated at 37 °C, for; 20; min and then at 51 °C for 20 min. Absorbance of the reaction mixture was measured at 660 nm. As a standard drug loprin was used instead of the plant samples. The test was done in triplicate and percentage inhibition of protein denaturation was calculated by following; formula:

$$ \mathrm{Inhibition}\  \mathrm{of}\  \mathrm{denaturation}\%=\frac{Abs\  Control- Abs\  sample}{Abs\  control}\times 100 $$

### Animal studies

Animals of six weeks old (180–200 g) Sprague-Dawley female and male rats-were maintained at 24 ± 3 °C with a 12-h dark/light cycle-at Primate Facility of Quaid-i-Azam University Islamabad, Pakistan. The animals were well fed and were sustained in standard laboratory conditions. The animals were bred with basal diet with water ad libitum and were sustained in standard laboratory conditions.

#### Acute toxicity studies

Sprague-Dawley female rats (*Rattus novergicus*) of 150–180 g were arbitrarily distributed in 30 groups each contained three rats. Rats of each group were orally administered SMM, SMH, SMC, SME, SMB and SMA at doses of 250 mg/kg, 500 mg/kg, 1000 mg/kg, 2000 mg/kg and 3000 mg/kg in the morning under fasting condition. The mortality and the abnormal behavior of the animals were observed after 30 min for 6 h then after 24 h for 15 days. At these doses the mortality and the and abnormal behavior was not detected so 50 mg/kg, 150 mg/kg and 300 mg/kg doses were selected for the evaluation of anti-inflammatory, analgesic and antipyretic activities. The guidelines of national Institute of Health, Islamabad were followed for use of animal care and experimentation and approved (Bch#0326) by the Institutional Ethical Committee of Quaid-i-Azam University Islamabad.

#### Anti-inflammatory activity

To ascertain the anti-inflammatory potential of the stem bark of *S. mukorossi* the carrageenan; induced; hind paw edema was followed in this study [49]. Anti-inflammatory activity was performed on Sprague-Dawley male rats of 150–200 g weight. Rats were randomly divided in to 20 groups with seven animals in each and normal paw volume of each rat was measured before the experiment. Group I was treated with 1% saline and Group II was treated with diclofenac potassium (10 mg/kg). Animals of all other groups were treated with SMM, SMH, SMC, SME, SMB and SMA at doses of 50 mg/kg, 150 mg/kg and 300 mg/kg in the morning. All these doses were administered 30 min earlier before the sub-plantar injection of carrageenan (1 ml/kg; 1% in saline *w*/*v*) in hind paw. Paw volume was measured by digital plethysmometer immediately after carrageenan injection (zero hours) and was repeated after every one hour up to three hours. Percentage inhibition of edema was calculated for each animal by using the following formulae:

$$ {}^{"}\mathrm{EV} =^{"}{PVA}^{"}\hbox{-} {}^{"}{PVI}^{"} $$

Where, EV = Edema volume, PVI = Paw Volume = before injection of carrageenan (i.e. initial/paw/vol.), PVA = Paw volume’ after injection of carrageenan

$$ \mathrm{Edema}\  \mathrm{inhibition}\%=\frac{EVc- EVt}{EVc}\times 100 $$

#### Anti-pyretic activity

Anti-pyretic activity of the plant samples was estimated by using the protocol of Afsar et al. [50]. Rectal temperature of each animal was checked by digital thermometer. Rats were subcutaneously injected at 10 ml/kg with 20% aqueous suspension of brewer’s yeast (*Saccharomyces cerevisiae*) to induce the pyrexia and have access to water only for 24 h. Rats showing rectal temperature at least 1 °C higher from normal were included in the evaluation of anti-pyretic activity. Pyrexia induced Sprague-Dawley male rats were arranged in 20 groups with 7 rats in each. Animals of Group I received 10 ml/kg of 10% DMSO, Group II received 10 mg/kg of paracetamol as standard drug, whereas all other groups received SMM, SMH, SMC, SME, SMB and SMA at doses of 50 mg/kg, 150 mg/kg and 300 mg/kg in 10% of DMSO in the morning. The rectal temperature of all the animals was recorded after 1 h, 2 h, 3 h and 4 h of the administration of plant samples.

#### Analgesic activity

Protocol of Muhammad et al. [49] was followed to evaluate the analgesic activity of plant samples. Sprague Dawley male rats were tested for analgesia screening and the rats showing greater than 15 s of latency time on a hot plate (Harvard apparatus) maintained at −50 ± 0.1 °C were excluded. Animals were divided in to 20 groups each having 7 rats. Rats of Group I received 10 ml/kg of 10% DMSO while animals of Group II received 10 mg/kg of loprin a standard drug. Animals of other groups received SMM, SMH, SMC, SME, SMB and SMA at doses of 50 mg/kg, 150 mg/kg and 300 mg/kg in the morning. The induction of analgesia (time in seconds for which rat-remained on the hot plate without licking or flicking of hind-limb or jumping) was recorded at 0, 30, 60 and 120 min after the administration of the plant samples and drug. In order to-avoid tissue-damage, cut off-time of 20 s was set-for all animals. Percent analgesia-was calculated using the following formula.

$$ \mathrm{Analgesia}\%=\frac{Tf- Ti}{Ti}\times 100 $$

### Statistical analysis

All the data presented as mean ± SD. The in vitro studies comprised of three replications while animal studies were conducted on seven animals. The in vitro activities obtained were analyzed by GraphPad Prism 5 for the determination of IC_50_ and correlation. For in vivo studies the consequences of different treatments given to animals were evaluated by one way analysis of variance through Statistix 8.1. Level of significance was observed at *p* ≤ 0.05 for correlation studies and *p* ≤ 0.01 for in vivo studies.

## Results

### Extraction yield

Dry powder 2 kg of *S. mukorossi* stem bark was soaked in crude methanol for four weeks and provided 93 g yield of SMM. From this 50 g was fractionated with various organic solvents having escalating polarity in order of SMH (18.36 g), SMC (1.7 g), SME (4.992 g) and SMB (5.9 g). Residue soluble fraction known as aqueous fraction (SMA) gave a yield of 5.9 g.

### Determination of total phenolic and flavonoid content

The total phenol and flavonoid contents in carious extract/fractions of stem bark of *S. mukorossi* are shown in Table 1. SME showed the maximum quantity of total phenolics (594.16 ± 4.3 mg GAE/g) followed by SMM (562.17 ± 4.77 mg GAE/g), SMC (444.3 ± 3.9 mg GAE/g), SMB (357 ± 4.3 mg GAE/g) and SMA (252.5 ± 2.41 mg GAE/g). However, SME constituted the maximum quantity of flavonoids (96.9 ± 3.2 mg RE/g) followed by SMM (55.5 ± 3.2 mg RE/g), SMC (52.8 ± 2.01), SMB (37.8 ± 1.9 mg RE/g) and SMA (11.02 ± 1.3 mg RE/g).

**Table 1:** Total flavonoid and phenolic contents and other metabolites in stem bark of *S. mukorossi*

Plant sample	Total flavonoid content (mg RUE/g)	Total phenolic content (mg GAE/g)
SMM	55.5 ± 3.2^b^	562.17 ± 4.77^b^
SMH	–	–
SMC	52.8 ± 2.01^b^	444.33 ± 3.9^c^
SME	96.9 ± 3.2^a^	594.16 ± 4.3^a^
SMB	37.8 ± 1.9^c^	357 ± 4.3^d^
SMA	11.02 ± 1.3^d^	252.3 ± 2.41^e^

Each value is represented as mean ± SD (n = 3). Means with different superscript (^a-e^) letters in the rows are significantly (P < 0.01) different from one another. SMM, *S. mukorossi* methanol extract of stem bark; SMH, n-hexane fraction of SMM; SMC, chloroform fraction of SMM; SME, ethyl acetate fraction of SMM; SMA, soluble residual aqueous fraction of SMM; −, absent

### In vitro antioxidant activities

#### DPPH radical scavenging activity

**Fig. 1:**
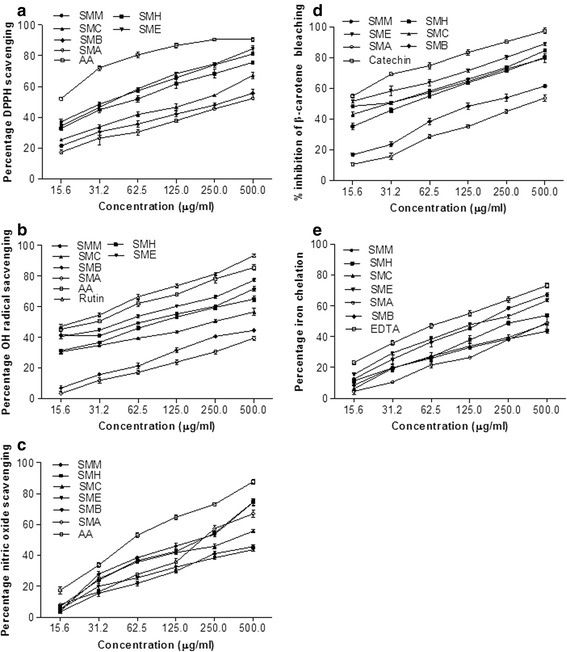
In vitro antioxidant activities of the methanol extract of the stem bark of *S. mukorossi* and its derived fractions. (**a**) DPPH scavenging activity; (**b**) hydroxyl radical scavenging activity; (**c**) nitric oxide scavenging activity; (**d**) inhibition of carotene bleaching assay; (**e**) iron chelation activity. SMM, *S. mukorossi* methanol extract of stem bark; SMH, n-hexane fraction of SMM; SMC, chloroform fraction of SMM; SME, ethyl acetate fraction of SMM; SMA, soluble residual aqueous fraction of SMM; AA, ascorbic acid. Data represent mean (3) ± SD

DPPH radical scavenging activity of all the extract/fractions is given in Table 2. SMM deliberated the highest scavenging activity (IC_50_ = 162.5 ± 2.29 μg/ml) among the extract/fractions of the stem bark of *S. mukorossi*. The IC_50_ value recorded for DPPH radical scavenging of ascorbic acid was 89.2 ± 4.05 μg/ml. Based on the IC_50_ values for DPPH radical scavenging the extract/fractions of *S. mukorossi* bark can be arranged in order of SMM > SME > SMH > SMA > SMC > SMB. The extract/fractions exhibited a dose dependent response for the DPPH radical scavenging activity (Fig. 1).

**Table 2:** IC_50_ values (μg/ml) of different antioxidant activities of *S. mukorossi*

Plant sample	DPPH radical scavenging activity (μg/ml)	Hydroxyl radical scavenging activity (μg/ml)	Nitric oxide scavenging activity (μg/ml)	β-Carotene bleaching inhibition activity (μg/ml)	Iron chelating activity (μg/ml)
SMM	162.5 ± 1.29^d^	61.97 ± 7.56^e^	161.9 ± 5.33^e^	24.97 ± 4.9^d^	162.5 ± 8.3^d^
SMH	304.9 ± 4.5^c^	101.3 ± 3.88^d^	281.4 ± 6.12^c^	44.67 ± 4.2^c^	304.9 ± 9.9^c^
SMC	530.7 ± 5.7^b^	239.6 ± 8.44^c^	207.6 ± 5.8^d^	30.5 ± 2.5^d^	530.7 ± 6.5^b^
SME	173.2 ± 1.01^d^	44.7 ± 3.09^f^	152.9 ± 6.7^e^	16.24 ± 5.1^e^	173.2 ± 5.6^d^
SMB	718.8 ± 9.47^a^	578.7 ± 3.7^b^	531.2 ± 4.7^b^	182.3 ± 5.8^b^	718.8 ± 9.7^a^
SMA	523.7 ± 8.45^b^	939.6 ± 4.44^a^	653.7 ± 4.2^a^	355.5 ± 7.8^a^	523.7 ± 5.9^b^
Rutin	**–**	21.35 ± 3.71^g^	**–**	**–**	**–**
AA	89.2 ± 4.05^e^	25.93 ± 3.08^g^	65.92 ± 1.99^f^	**–**	**–**
EDTA	**–**	**–**	**–**	**–**	89.2 ± 5.99^e^
Catechin	**–**	**–**	**–**	11.8 ± 3.6^e^	**–**

Values are presented as mean ± SD (n = 3). Means with different superscript (^a-e^) letters in the rows are significantly (P *<* 0.01) different from one another. AA, Ascorbic acid; SMM, *S. mukorossi* methanol extract of stem bark; SMH, n-hexane fraction of SMM; SMC, chloroform fraction of SMM; SME, ethyl acetate fraction of SMM; SMA, soluble residual aqueous fraction of SMM; −, not determined

#### Hydroxyl radical scavenging activity

The results of this study indicated the dose dependent pattern for the scavenging of hydroxyl radicals of all the extract/fractions of *S. mukorossi* bark (Fig. 1). Lowest IC_50_ value was shown by SME 44.7 ± 3.09 μg/ml followed by IC_50_ value of 61.97 ± 7.56 μg/ml for the SMM. However, the highest IC_50_ value was observed for SMA (939.6 ± 14.44 μg/ml). IC_50_ values of SMB, SMH, and SMC were significantly different from standard the IC_50_ value of rutin (21.35 ± 1.71 μg/ml). Overall the pattern of IC_50_ values for the scavenging of hydroxyl radical was exhibited as SME < SMM < SMH < SMC < SMB < SMA (Table 2).

#### Nitric oxide scavenging activity

Dose dependent pattern for the nitric oxide scavenging potential has been exhibited by the extract/fractions of the stem bark of *S. mukorossi* in the present study (Fig. 1). SME distinctly showed the scavenging ability for nitric oxide and the lowest IC_50_ value (152.9 ± 6.7 μg/ml) was recorded and it was followed by SMM (161.9 ± 5.33 μg/ml). The IC_50_ value of 65.92 ± 1.99 μg/ml was recorded for ascorbic acid for the scavenging of nitric oxide. The SMA exhibited the lowest scavenging activity for nitric oxide with IC_50_ value of 653.70 ± 4.2 μg/ml. The IC_50_ value for other extracts was; 281.4 ± 6.12 μg/ml (SMH), 207.6 ± 5.8 μg/ml (SMC) and 531.2 ± 4.7 μg/ml (SMB) as shown in Table 2.

#### β-carotene bleaching inhibition activity

In this study the maximum inhibition of β-carotene bleaching activity was exhibited by SME (IC_50_ = 16.24 ± 5.1 μg/ml) followed by SMM with IC_50_ value of 24.97 ± 4.9 μg/ml. The IC_50_ value determined for SMC was 30.5 ± 2.5 μg/ml followed by SMH (44.67 ± 4.2 μg/ml) and SMB (182.3 ± 5.8 μg/ml). However, the SMA showed the lowest inhibition of bleaching activity with IC_50_ values of 653.7 ± 4.20 μg/ml compared with standard catechin having IC_50_ value of 11.86 ± 3.6 μg/ml (Table 3). The inhibition of bleaching power pattern of various extract/fractions is shown in Fig. 1.

**Table 3:** Correlation of IC_50_ values of different antioxidant activities with total phenolic and total flavonoid contents

Antioxidant activity	TFC	TPC
DPPH radical scavenging activity	0.768^*^	0.9338^***^
Hydroxyl radical scavenging activity	0.2861	0.6685^*^
Iron chelating assay	0.8434^**^	0.928^***^
Nitric oxide scavenging activity	0.7935^*^	0.8966^**^
Inhibition of β-carotene bleaching	0.883^**^	0.8472^**^
Phosphomolybdenum assay	0.7639^*^	0.8648^**^
Reducing power assay	0.6725^*^	0.9055^**^

^*,**^indicate significance at *P* < 0.05 and P < 0.01, respectively

#### Iron chelation activity

The present study indicated the dose dependent pattern of iron chelating activity of the methanol extract and the derived fractions of *S. mukorossi* bark (Fig. 1). SMM showed the lowest IC_50_ value of 162.5 ± 8.3 μg/ml followed by SME (173.2 ± 5.6 μg/ml) and SMH (304.9 ± 9.9 μg/ml). However SMB exhibited the lowest potential for iron chelation with IC_50_ value of 718.8 ± 9.7 μg/ml.

#### Reducing power assay

**Fig. 2:**
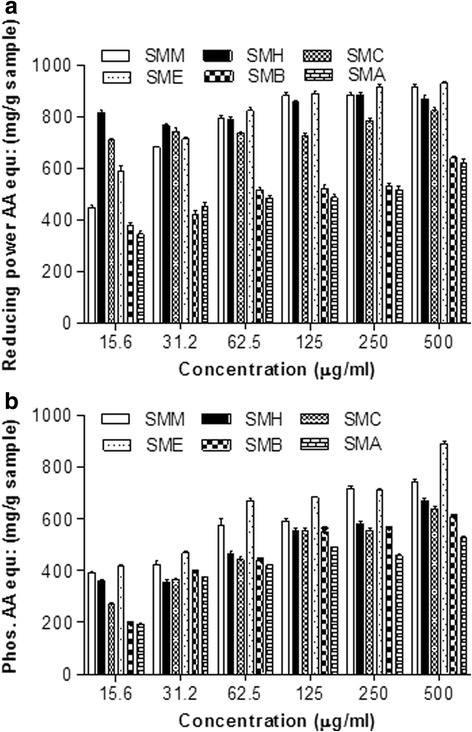
(**a**) Reducing power activity determined as ascorbic acid equivalent; (**b**) Total antioxidant activity determined as ascorbic acid equivalent. Data represent mean (3) ± SD

The results of the present study showed the dose dependent response for reducing power of the various extract/fractions of *S. mukorossi* bark and the results were expressed as ascorbic acid equivalent mg/g sample (Fig. 2). In this study the SME at 250 μg/ml concentration exhibited the highest reducing power (903.22 mg ascorbic acid equivalent/g). The reducing power of the other extract/fractions at 250 μg/ml concentration was; SMM (881.72 mg ascorbic acid equivalents/g), SMH (870.96 mg ascorbic acid equivalents/g sample), SMC (783.96 mg ascorbic acid equivalents/g sample), SMB (522.97 mg ascorbic acid equivalents/g sample) and SMA (503.42 mg ascorbic acid equivalents/g sample).

#### Phosphomolybdenum activity

Various extract/fractions of the stem bark of *S. mukorossi* established a dose dependent total antioxidant capacity and the results were expressed as ascorbic acid equivalent mg/g sample (Fig. 2). SMM at 250 μg/ml concentration exhibited the maximum total antioxidant capacity (717.12 ascorbic acid equivalents/g sample) followed by SME (703.36 mg ascorbic acid equivalents/g sample).

#### Correlation studies

Multiple antioxidant assays were executed to find the IC_50_ values (μg/ml) and their correlation with the total phenolic and flavonoid content was determined. All assays except OH radical scavenging showed positive and significant correlation with the total phenolic and flavonoid content. Strong positive association of the total phenolic content with DPPH and iron chelation activity (*P* < 0.001) was established while the iron chelation activity and inhibition of β-carotene bleaching activity showed strong association (*P* < 0.01) with total flavonoid content (Table 3).

### Inhibition of heat induced protein denaturation activity

**Fig. 3:**
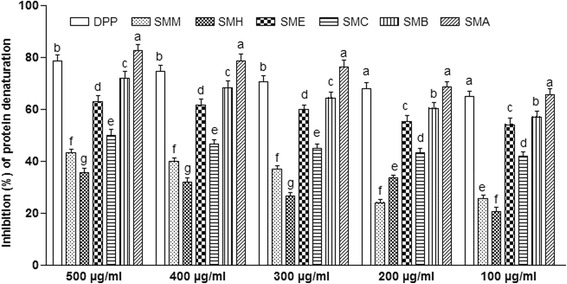
Percent inhibition of protein denaturation at different concentrations of *S. mukorossi* extract/fraction. Each bar represents as Mean ± SD (*n* = 3). Superscript (^a-f^) indicate significance for specific concentration at *P* < 0.01. SMM, *S. mukorossi* methanol extract of stem bark; SMH, n-hexane fraction of SMM; SMC, chloroform fraction of SMM; SME, ethyl acetate fraction of SMM; SMA, soluble residual aqueous fraction of SMM

The SMM and its derived fractions (SMH, SMC, SME, SMB and SMA) were evaluated for in vitro anti-inflammatory activity through inhibition of heat induced protein denaturation assay (Fig. 3). All extract/fractions exhibited dose dependent inhibition of heat induced protein denaturation and the IC_50_ (μg/ml) values were found in order; SMA > SMB > SME > SMC > SMM > SMH. The range of percent inhibition of SMA varied between 65 and 82%; SMB (57–72%); SME (54–63%); SMC (42–50%); SMM (26–43%) and SMH (21–36%) at concentration range of 100 μg/ml to 500 μg/ml. The standard drug loprin exhibited IC_50_ value of 23 μg/ml and percent inhibition varied between 65 and 79% at concentration range of 100 μg/ml to 500 μg/ml.

### In vivo bioassays

#### Anti-inflammatory activity

**Fig. 4:**
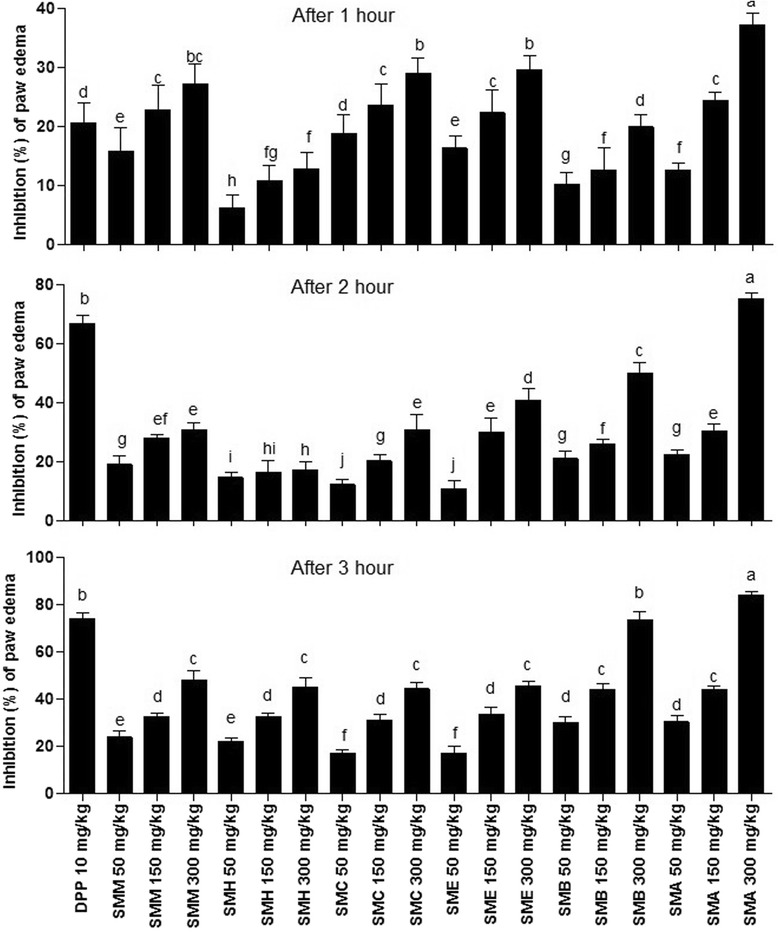
Percent inhibition of carrageenan induced paw edema in rat. Each bar represents as Mean ± SD (n = 3). Superscript (^a-j^) indicate significance at P < 0.01. DPP, diclofenac potassium; SMM, *S. mukorossi* methanol extract of stem bark; SMH, n-hexane fraction of SMM; SMC, chloroform fraction of SMM; SME, ethyl acetate fraction of SMM; SMA, soluble residual aqueous fraction of SMM

Carrageenan induced paw edema assay was used to determine the anti-inflammatory activity of plant samples (Fig. 4). Data obtained indicated a time and concentration dependent anti-inflammatory activity of the extract/fractions of the stem bark of *S. mukorossi*. Percent edema inhibition by the treatment of diclofenac potassium (standard drug) recorded after 1 h, 2 h and 3 h was 20.66 ± 3.37%, 66.7 ± 2.85% and 73.99 ± 2.54%, respectively. Among the extract/fractions SMM, SMH, SMC and SME at 300 mg/kg exhibited (48 ± 4.14%, 45 ± 3.92, 44.30 ± 2.59 and 45.41 ± 2.11%, respectively) the inhibition of paw edema after 3 h of treatment. However, SMB and SMA at 300 mg/kg strongly inhibited the edema development (73.43 ± 3.70%, 84.19 ± 1.48%) after 3 h in rat. After 3 h of carrageenan treatment different fractions exhibited the inhibition of edema in the order SMA > SMB > SMM > SME > SMH and SMC at 300 mg/kg concentration.

#### Analgesic activity

**Fig. 5:**
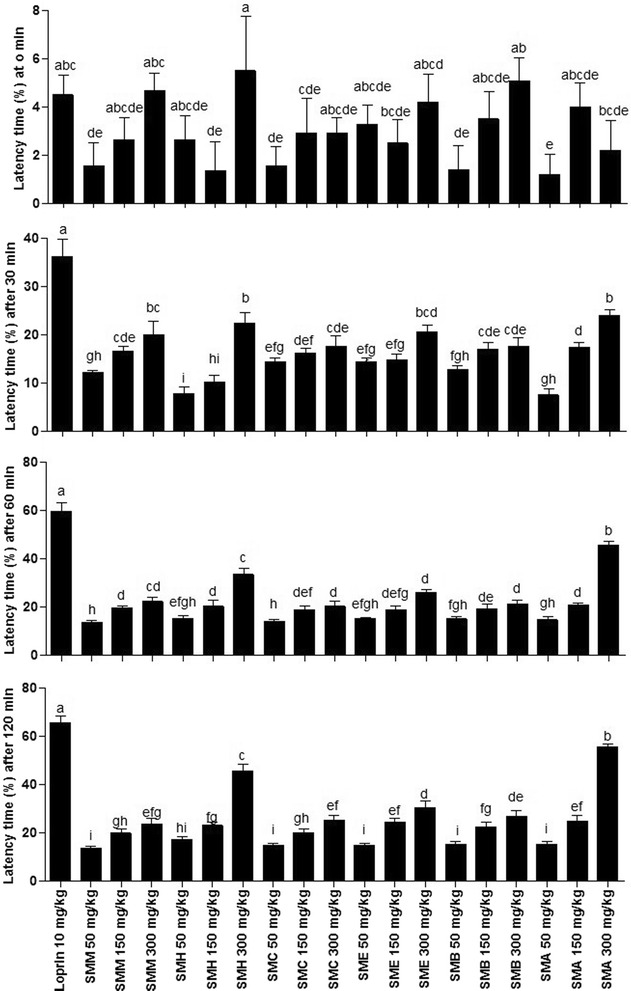
Percent analgesia induced with different extract/fractions of *S. mukorossi* in rat. Each bar represents as Mean ± SD (n = 3). Superscript (^a-j^) indicate significance at *P* < 0.01. SMM, *S. mukorossi* methanol extract of stem bark; SMH, n-hexane fraction of SMM; SMC, chloroform fraction of SMM; SME, ethyl acetate fraction of SMM; SMA, soluble residual aqueous fraction of SMM

Hot plate procedure was adopted for the estimation of analgesic activity of different extract/fractions of the stem bark of *S. mukorossi*. All the samples have displayed increase in latency time at 0, 30, 60 and 120 min with increase of concentration as shown in Fig. 5. Administration of SMA at 300 mg/kg showed strong analgesic activity with percent analgesia of 2.21 ± 1.24, 23.97 ± 1.28, 45.64 ± 1.66 and 55.78 ± 1.22 in comparison to standard loprin drug (10 mg/kg) with percent latency time of 4.5 ± 0.81, 36.35 ± 2.56, 59.64 ± 3.68, 65.64 ± 2.76 at 0, 30, 60 and 120 min, respectively.

#### Anti-pyretic activity

**Fig. 6:**
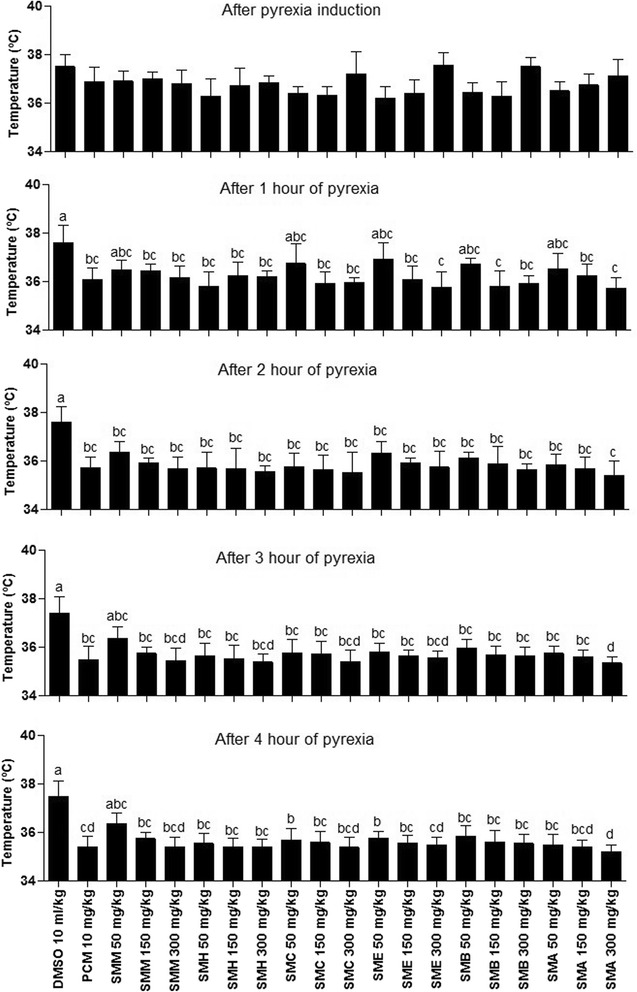
Antipyretic potential of different extract/fractions of *S. mukorossi* in rat. Each bar represents as Mean ± SD (n = 3). Superscript (^a-d^) indicate significance at P < 0.01. SMM, *S. mukorossi* methanol extract of stem bark; SMH, n-hexane fraction of SMM; SMC, chloroform fraction of SMM; SME, ethyl acetate fraction of SMM; SMA, soluble residual aqueous fraction of SMM

Antipyretic effect of various extract/fractions of the stem bark of *S. mukorossi* was assessed in rat. SMA at 300 mg/kg exhibited the strong antipyretic activity and decreased the rectal temperature from 37.11 ± 0.71 °C to 35.21 ± 0.41 °C after 4 h of administration to rats. The decrease of rectal temperature from 37.5 ± 0.4 °C to 35.54 ± 0.38 °C was recorded for SMB at 300 mg/kg after 4 h of administration to rat. Other extract/fractions also decreased the rectal temperature on concentration and on time dependent manner (Fig. 6).

## Discussion

A wide variety of pathological demonstrations indicated the substantial involvement of free radicals. Antioxidants either scavenge the reactive oxygen species or reduce the free radicals [1]. Low levels of antioxidants in the living system assist the development of ageing related diseases such as atherosclerosis, cancers, diabetic neuropathy and Alzheimer’s disease. An anticipated solution to such problem is the enhancement of antioxidant that is present in plants [7]. There is a complex mechanism of scavenging free radicals inside human body and consists of a group of enzymes. Consequently different assays have been planned to get a complete picture of antioxidant potential of the stem bark extract of *S. mukorossi*.

Bleaching of DPPH solution is extensively used to estimate the electron donating aptitude of natural products. The electron donating capacity and concentration of the active constituent in natural product contributes towards the free radical scavenging activity [8]. In the current study SMM and SME illustrate extensively scavenging ability and were significantly correlated with TPC suggested the presence of hydrogen donating constituents. Sodium nitro-prusside reacts with oxygen and nitrite is formed which is the basic process for generation of nitrite in nitric oxide radical scavenging assay. Antioxidant present in plant extracts competes for oxygen with nitric oxide and in this way inhibits the formation of nitrite. SME inhibited nitric oxide generation with the lowest IC_50_ value of 152.9 ± 6.7 μg/ml among the extract/fractions. In this study significant correlation was observed between nitric oxide radical scavenging activities and with TPC and TFC indicating the existence of primary antioxidant constituents in SME and thus prevents damages in biological tissues [50].

Hydrogen peroxide is present at low level in the biological system and is catalyzed in to water and oxygen. During this process hydroxyl radicals are generated and considered the most damaging species in the biological system which causes cellular injuries by its damaging action on proteins, DNA and lipids [51]. The hydroxyl radical is considered as a damaging species in pathophysiological process and it is able to damage almost each molecule of biological system and contribute to carcinogenesis, mutagenesis and cytotoxicity [52]. Hydroxyl radicals are formed by the reaction between H_2_O_2_ and the ferrous that would reacts with 2-deoxyribose. This reaction can be blocked by adding thiobarbituric acid that would produce red color if the malonaldehyde produced as a result of the reaction between the free radical and 2-deoxyribose. Hydroxyl radicals scavenging capacity of an extract is directly proportional to its antioxidant activity that is illustrated by the low intensity of red color. All fractions of *S. mukorossi* when added to the reaction mixture vigorously scavenged the hydroxyl radicals and inhibited the degradation of 2-deoxyribose. Hydrogen peroxide occurs naturally at low concentration in air, water, human body, plants, microorganisms and food. H_2_O_2_ is quickly decayed into water and oxygen and this may create hydroxyl radicals (•OH) that may begin lipid peroxidation and consequently induce DNA damages [53]. SME significantly scavenged hydrogen peroxide that can be suggested to the presence of phenolic compounds that could contribute electrons to hydrogen peroxide, thus neutralizing it into water.

Another significant system of antioxidant activity includes chelation of metal ions. Antioxidants that act as chelating agents are able to diminish or stabilize the oxidized metal ions. Iron chelating data illustrate that different fractions may have the capacity to decrease the oxidative damage by chelating iron ions that may otherwise contribute in breakdown of metal catalyzed hydrogen peroxide and Fenton-Type reactions. The iron sequestering efficacy of different extract/fractions deliberated at different concentrations (15.6–1000 μg/ml) suggest the presence of endogenous chelating mediators like phenolics and flavonoids. Additionally, some phenolic compounds having well oriented functional groups that hold the capability to defend adjacent to oxidative damage by chelating metal ions [54].

The efficacy of plant fractions to impede oxidation of linoleic acid emulsion is a sign of complex composition of extract/fractions to cooperate with emulsion components. The present data recommended that the extract/fractions have a prominent tendency to scavenge free radicals that result in more stable non-reactive substances and to cease radical chain reactions. β-carotene bleaching inhibition was accounted in diverse solvent extracts of *Jurinea dolomiaea* roots [55].

In reducing power assay SME showed the highest reducing power ability at 250 μg/ml followed by SMM and SMH with IC_50_ values of 903.22, 881.72 and 870.96 mg of ascorbic acid equivalents/g sample, respectively. Phosphomolybdate is an additionally important in vitro antioxidant assay to assess the total antioxidant capacity of the plant extract. The assay principal follows the conversion of Mo(VI) to Mo (V) by extract or the compound which possess antioxidant potential and result in green phosphate Mo (V). The electron/hydrogen donating pattern of antioxidants depends upon its structure and series of redox reactions occurring in the activity. Recent study shows that SME possesses strong total antioxidant capacity that suggests the protective potential of SME in biological system [55]. The antioxidant activities recorded in this study might be due to the presence of antioxidant compounds stigmasterol, oleanolic acid, luteolin, luteolin 8-퐶-훽-glucoside (orientin), luteolin 6-퐶-훽-glucoside (isoorientin), luteolin 7-푂-훽-glucuronide and rutin [15].

To the best of our knowledge this is the first report on antipyretic, analgesic and anti- inflammatory activities of the stem bark of *S. mukorossi* extract/fractions. Tissue protein denaturation is a well-documented base of arthritic and inflammatory diseases in which secondary structure and tertiary structure of the proteins are lost by external stress or compounds such as strong acid/base, inorganic salt, organic solvent or high temperature. Mostly biological proteins when denatured lose their biological functioning. High temperature causes denaturation of BSA (bovine serum albumin) and this denatured protein exhibits the antigens similar to the Type III hyper-sensitive reactions. Heat denatured proteins causes delayed hypersensitivity which is associated to diseases like glomerulo-nephritis; serum disease and rheumatoid arthritis [56]. In present research various extract/fractions of *S. mukorossi* have revealed effective anti-inflammatory responses in a concentration dependent fashion similar to earlier reports [57]. These results suggest that the *S. mukorossi* extract/fractions can be evaluated for anti-inflammatory effects.

Carrageenan tempted inflammation is a frequently used method for evaluating anti-inflammatory influence of composites or natural products. Most probable mode of action of carrageenan persuaded inflammation is bi-phasic. Initial phase (0–1 h) of edema is attributed by the release of histamine, 5-hydroxytryptamine and bradykinin; and is not inhibited by the use of NSAIDs such as indomethacin or aspirin [3]. During the 2nd phase (1–6 h) of edema development elevated level of prostaglandins and inducible cyclooxygenase (COX-2) has been demonstrated in hind paw edema of rat [58]. Time dependent increase in edema, nitrite/nitrate and prostaglandins E2 (PGE2) has been characterized in the exudate. Infiltration of localneutrophils in the inflamed area plays their part for the development of inflammation by producing the reactive oxygen species (ROS) and most importantly the superoxide and hydroxyl radicals. Another mediator of inflammation nitric oxide (NO) is produced by the cytokine-inducible, calcium/calmodulin-independent isoform of nitric oxide synthase (iNOS). As a consequence of NO synthesis vascular permeability and flow of blood increased towards the inflamed site and also increase the production of pro-inflammatory cytokines. Simultaneous occurrence of NOS and COX pathway exaggerated the inflammation response. The inhibition ofinflammation during the early and the late phase of edema with SMA suggest that it might have mediated the anti-inflammatory activity by inhibiting the release of mediators of inflammation [2, 58].

Hot plate test (thermal stimulation method) is a well-documented process for assessing centrally acting analgesic around spinal reflexes that comprises pain transmission from periphery via nociceptors to the brain through spinal cord. Such actions have been attributed by the inhibition of phospholipase A2 and in that way hindering the breakdown of arachidonic acid which in turn inhibits the pain producing prostaglandins. Diclofenac sodium prompts analgesic activity by opioid receptors activation and the resemblance between the effects of extracts with reference diclofenac sodium, shows that they might work analogous to diclofenac sodium to decrease pain sensation [58]. Anti-inflammatory and analgesic effects of the extracts especially the SMA from stem bark of *S. mukorossi* are promising.

Antipyretic activity of plant extracts and drugs can be evaluated by subcutaneous injection of Brewer’s yeast induced pyrexia in animal models. During this process production of prostaglandins is increased and the inhibitory potential of plant extracts/drugs on prostaglandins synthesis can be considered as a useful test for anti-pyretic potential [2]. In this connection significant antipyretic activity of SMM and its fraction SMA at 300 mg/kg dose has been recorded and their antipyretic activity was similar to the standard drug paracetamol. As paracetamol exert its antipyretic activity by blocking the cyclooxygenase activity thereby inhibiting the synthesis of prostaglandins. The observed anti-inflammatory, anti-nociceptive and antipyretic effects of SMM and its derived fractions in this study might be attributed by the presence of pharmacologically active metabolites. The inflammatory process is a complex mechanism comprising several biochemical events it may therefore be worthwhile to investigate the exact point in the biochemical events where the extract exerts its anti-inflammatory effect.

## Conclusion

Results of this study suggested that the presence of polyphenols and flavonoids and other constituents in polar extract/fractions might alleviate the inflammation and pain inducing mediators. This study endorsed the use of bark of *S. mukorossi* by the local communities for the disorders related to inflammation.

## Abbreviations

AA: Ascorbic acid
ROS: Reactive oxygen species
SMA: Soluble residual aqueous fraction of SMM
SMC: Chloroform fraction of SMM
SME: Ethyl acetate fraction of SMM
SMH: N-hexane fraction of SMM
SMM: *S. mukorossi* methanol extract of stem bark
TFC: Total flavonoid content
TPC: Total phenolic content

## References

[1] Shah NA, Khan MR (2017). Increase of glutathione, testosterone and antioxidant effects of *Jurenia dolomiaea* on CCl_4_ induced testicular toxicity in rat. BMC Complement Altern Med.

[2] Jan S, Khan MR (2016). Antipyretic, analgesic and anti-inflammatory effects of *Kickxia ramosissima*. J Ethnopharmacol.

[3] Younis T, Khan MR, Sajid M, Majid M, Zahra Z, Shah NA (2016). *Fraxinus xanthoxyloides* leaves reduced the level of inflammatory mediators during *in vitro* and *in vivo* studies. BMC Complement Altern Med.

[4] Harirforoosh S, Asghar W, Jamali F (2014). Adverse effects of nonsteroidal anti-inflammatory drugs: an update of gastrointestinal, cardiovascular and renal complications. J Pharm Pharmaceut Sci.

[5] Afsar T, Razak S, Khan MR, Almajwal A (2017). Anti-depressant and anxiolytic potential of *Acacia hydaspica* R. Parker aerial parts extract: modulation of brain antioxidant enzyme status. BMC Complement Altern Med.

[6] Vane JR, Botling RM (1995). New insights into the mode of action of anti-inflammatory drugs. Inflam Res.

[7] Rashid U, Khan MR (2017). *Fagonia olivieri* prevented hepatorenal injuries induced with gentamicin in rat. Biomed Pharmacother.

[8] Tahir I, Khan MR, Shah NA, Aftab M (2016). Evaluation of phytochemicals, antioxidant activity and amelioration of pulmonary fibrosis with *Phyllanthus emblica* leaves. BMC Complement Altern Med.

[9] Kirtikar KR, Basu BD (1991). Indian medicinal plants.

[10] Dhar JP, Bajpai VK, Setty BS, Kamboj VP (1989). Morphological changes in human spermatozoa as examined under scanning electron microscope after *in vitro* exposure to saponins isolated from *Sapindus mukorossi*. Contraception.

[11] Albiero MLA, Bacchi ME (2001). M. S. K. Mourao MSK., Caracterizacao anatomica das folhas, frutos e sementes de *Sapindus saponaria* L., (Sapindaceae). Acta Sci.

[12] Sonawane SM, Sonawane H (2015). A review of recent and current research studies on the biological and pharmalogical activities of *Sapindus mukorossi*. Int J Interdis Res Innov.

[13] Francis G, Kerem Z, Makkar HPS, Becker K (2002). The biological action of saponins in animal systems: a review. Brit. J Nutr.

[14] Abdel-Wahab MS, Selim AM (1985). Lipids and flavonoids of *Sapindus saponaria*. Fitoterapia.

[15] Rashed KN, Ćirić A, Glamočlija J, Calhelha RC, Ferreira ICFR, Soković M (2013). Antimicrobial activity, growth inhibition of human tumour cell lines, and phytochemical characterization of the hydromethanolic extract obtained from *Sapindus saponaria* L. aerial parts. Biomed Res Int.

[16] Ibrahim M, Anjum A, Waheed MA (2012). Curative effect of extracts of *Sapindus mukorossi* and *Rheum emodi* in CCl_4_ induced liver cirrhosis in male rats. Global. J Med Res.

[17] Verma N, Amresh G, Sahu PK, Mishra N, Singh AP (2012). Rao ChV. Antihyperglycemic activity, antihyperlipedemic activity, haematological effects and histopathological analysis of *Sapindus mukorossi* Gaerten fruits in streptozotocin induced diabetic rats. Asian Pac J Trop Med.

[18] Jedage D, Manjunath KP (2016). Phytochemical, pharmacological evaluation of *Sapindus emarginatus* Vahl. Bark extract for nephroprotective activity. Int J Pham Sci Res.

[19] Chakraborty A, Amudha P, Geetha M, Singh NS (2010). Evaluation of anxiolytic activity of methanolic extract of *Sapindus mukorossi* Gaertn. In mice. Int J Pharma and Bio Sci.

[20] Anitha K, Jyothi Y, Veena AV, Bora D (2015). Evaluation of antiepileptic activity of fruit pericarp of *Sapindus mukorossi* in rats. J Chem Pharm Res.

[21] Chen C-Y, Kuo P-J, Chen Y-H, Huang J-C, Ho M-L, Lin R-J, Chang J-S, Wang H-M (2010). Tyrosinase inhibition, free radical scavenging, antimicroorganism and anticancer proliferation activities of *Sapindus mukorossi* extracts. J Taiwan Inst Chem Eng.

[22] Goli V, Gowrishankar NL, Macharla SP, Ramchander T, Bhaskar J, Bhaskar KV (2011). Effects of anti-inflammatory activity of *Sapindus mukorossi*. Int J Pharma Technol.

[23] Kumar CNS, Das A, Raj GRA (2014). Anti-inflammatory activity of *Sapindus laurifolius* leaf extract in wistar rats. J med plant. Studies.

[24] Arulmozhia DK, Sridhara N, Bodhankarb SL, Veeranjaneyulua A, Arora SK (2004). *In vitro* pharmacological investigations of *Sapindus trifoliatus* in various migraine targets. J Ethnopharmacol.

[25] Arulmozhi DK, Addepalli V, Bodhankar SL, Arora SK (2005). Effect of *Sapindus trifoliatus* on hyperalgesic *in vivo* migraine models. Braz J Med Biol Res.

[26] Takagi K, Park EH, Kato H (1980). Anti-inflammatory activities of hederagenin and crude saponin isolated from *Sapindus mukorossi* Gaertn. Chem Pharm Bull (Tokyo).

[27] Choi J, Jung HJ, Lee KT, Park HJ (2005). Antinociceptive and anti-inflammatory effects of the saponin and sapogenins obtained from the stem of *Akebia quinata*. J Med Food.

[28] Borgi W, Recio M-C, Rios JL, Chouchane N (2008). Anti-inflammatory and analgesic activities of flavonoid and saponin fractions from *Zizyphus lotus* (L.) lam. South Afr. J Bot.

[29] Arraua S, Delportea C, Cartagenaa C, Rodríguez-Díaz M, González P, Silva X, Cassels BK, Miranda HF (2011). Antinociceptive activity of *Quillaja saponaria* Mol. Saponin extract, quillaic acid and derivatives in mice. J Ethnopharmacol.

[30] Zhao J, Xu F, Huang H, Gu Z, Wang L, Tan W, He J, Chen Y, Li C (2013). Evaluation on anti-inflammatory, analgesic, antitumor, and antioxidant potential of total saponins from *Nigella glandulifera* seeds. Evidence-Based Complement Altern Med.

[31] Santos EN, Lima JCS, Noldin VF, Cechinel-Filho V, Rao VSN, Lima EF, Schmeda-Hirschmann G (2011). PTS, martins DTO. Anti-inflammatory, antinociceptive, and antipyretic effects of methanol extract of *Cariniana rubra* stem bark in animal models. An Acad Bras Cienc.

[32] Hassan HS, Sule MI, Musa MA, Emmanual AA, Ibrahim H, Hassan AS, Yaro AH (2011). Analgesic and anti-inflammatory activities of the saponin extract of *Carissa edulis* root in rodents. Int J biol biol. Chem Sci.

[33] Bazmi RR, Javed I, Salman UG, Razi MT, Rasool N (2014). Analgesic and anti-inflammatory activity of *Sophora mollis* (Leguminosae) leaves and stem extracts in mice. Int J Pharm Phytopharmacol Res.

[34] Hong-cheng LI, Ru-hua HU. Anti-inflammatory and analgesic effects of total saponins from *Aralia elate*. Chinese J Clin Rational Drug use. 2009;23

[35] Yao Y, Yang X, Shi Z, Ren G (2014). Anti-inflammatory activity of saponins from quinoa (*Chenopodium quinoa* Willd.) seeds in lipopolysaccharide-stimulated RAW 264.7 macrophages cells. J Food Sci.

[36] JS Y, Kim JH, Lee S, Jung K, Kim KH, Cho JY (2017). Src/Syk-targeted anti-inflammatory actions of triterpenoidal Saponins from Gac (*Momordica colchchinensis*) seeds. Am J Chin Med.

[37] Sharma A, Sati SC, Sati OP, Sati MD, Kothiyal SK (2013). Triterpenoid Saponins from the pericarps of Sapindus Mukorossi. J Chem.

[38] Upadhyay A, Singh DK (2012). Pharmacological effects of *Sapindus mukorossi*. Rev Inst Med Trop Sao Paulo.

[39] Kim D-O, Jeong SW, Lee CY (2003). Antioxidant capacity of phenolic phytochemicals from various cultivars of plums. Food Chem.

[40] Park Y-S, Jung S-T, Kang S-G, Heo BG, Arancibia-Avila P, Toledo F, Drzewiecki J, Namiesnik J, Gorinstein S (2008). Antioxidants and proteins in ethylene-treated kiwi fruits. Food Chem.

[41] Mensor LL, Menezes FS, Leitao GG, Reis AS, Dos Santos TC, Coube CS, Leitao SG (2001). 2001. Screening of Brazilian plant extracts for antioxidant activity by the use of DPPH free radical method. Phytother Res.

[42] Halliwell B, Gutteridge JMC, Aruoma OI (1987). 1987. The deoxyribose method: a simple “test-tube” assay for determination of rate constants for reactions of hydroxyl radicals. Anal Biochem.

[43] Leone AM, Francis PL, Rhodes P, Moncada S (1994). 1994. A rapid and simple method for the measurement of nitrite and nitrate in plasma by high performance capillary electrophoresis. Biochem Biophys Res Commun.

[44] Robinson NJ, Procter CM, Connolly EL, Guerinot MLA (1999). Ferric-chelate reductase for iron uptake from soils. Nature.

[45] Dapkevicius A, Venskutonis R, Van Beek TA, Linssen JPH (1998). 1998. Antioxidant activity of the extracts obtained by different isolation procedures from some aromatic herbs grown in Lithuania. J Sci Food Agri.

[46] Fejes S, Blazovics A, Lugasi A, Lemberkovics E, Petri G, Kery A (2000). *In vitro* antioxidant activity of *Anthriscus cerefolium* L. (Hoffm.) extracts. J Ethnopharmacol.

[47] Umamaheswari M, Chatterjee TK (2008). *In vitro* antioxidant activities of the fractions of *Coccinnia grandis* L. leaf extract. Afr J Trad Complement Altern Med.

[48] Sakat S, Juvekar AR, Gambhire MN (2010). *In vitro* antioxidant and anti-inflammatory activity of methanol extract of *Oxalis corniculata* Linn. Int J Pharm Pharma Sci.

[49] Muhammad N, Saeed M, Khan H (2012). Antipyretic, analgesic and anti-inflammatory activity of Viola Betonicifolia whole plant. BMC Complement Altern Med.

[50] Afsar T, Khan MR, Razak S, Ullah S, Mirza B (2015). Antipyretic, anti-inflammatory and analgesic activity of *Acacia hydaspica* R. Parker and its phytochemical analysis. BMC Complement Altern Med.

[51] Sajid M, Khan MR, Shah NA, Shah SA, Ismail H, Younis T, Zahra Z. 2016. Phytochemical, antioxidant and hepatoprotective effects of *Alnus nitida* bark in carbon tetrachloride challenged Sprague Dawley rats. BMC complement Altern med. 2016;16:268. 10.1186/s12906-016-1245-3 PMC4972964 27488054

[52] Bokhari J, Khan MR, Ul Haq I (2016). Assessment of phytochemicals, antioxidant and anti-inflammatory potential of *Boerhavia procumbens* banks ex Roxb. Toxicol Ind Health.

[53] Batool R, Khan MR, Majid M (2017). *Euphorbia dracunculoides* L. abrogates carbon tetrachloride induced liver and DNA damage in rats. BMC Complement Altern Med.

[54] Sahreen S, Khan MR, Khan RA, Shah NA (2013). Effect of *Carissa opaca* leaves extract on lipid peroxidation, antioxidant activity and reproductive hormones in male rats. Lipid Health Dis.

[55] Shah NA, Khan MR, Naz K, Khan MA (2014). Antioxidant potential, DNA protection, and HPLC-DAD analysis of neglected medicinal *Jurinea dolomiaea* roots. Biomed Res Int.

[56] Williams LAD, Connar AO, Ringer S, Whittaker JA, Conrad J, Voglar B, Rösner H (2008). The *in vitro* anti-denaturation effects induced by natural products and non-steroidal compounds in heat treated (immunogenic) bovine serum albumin is proposed as a screening assay for the detection of anti-inflammatory compounds, without the use of animals. West Indian Med J.

[57] Sajid M, Khan MR, Shah SA, Majid M, Ismail H, Maryam S, Batool R, Younis T (2017). 2017. Investigations on anti-inflammatory and analgesic activities of *Alnus nitida* Spach (Endl). Stem bark in Sprague Dawley rats. J Ethnopharmacol.

[58] Di Rosa M, Giroud JP, Willoughby DA (1971). Studies on the mediators of the acute inflammatory response induced in rats in different sites by carrageenan and turpentine. J Pathol.

